# Oral Administration of *Flavonifractor plautii* Strongly Suppresses Th2 Immune Responses in Mice

**DOI:** 10.3389/fimmu.2020.00379

**Published:** 2020-02-28

**Authors:** Tasuku Ogita, Yoshinari Yamamoto, Ayane Mikami, Suguru Shigemori, Takashi Sato, Takeshi Shimosato

**Affiliations:** ^1^Department of Biomolecular Innovation, Institute for Biomedical Sciences, Shinshu University, Nagano, Japan; ^2^Graduate School of Integrated Sciences for Life, Hiroshima University, Higashi-Hiroshima, Japan; ^3^Department of Biomedical Engineering, Graduate School of Science and Technology, Shinshu University, Nagano, Japan

**Keywords:** *Flavonifractor plautii*, Th2, MLN, Deferribacteres, microbiota

## Abstract

The bacterium *Flavonifractor plautii* (FP), which is found in human feces, has been reported to participate in catechin metabolism in the gut, but this bacterium's effects on immune function are unclear. We assessed the effect of oral administration of FP on the immune response in ovalbumin (OVA) -sensitized mice. We demonstrated that the FP treatment suppressed interleukin (IL)-4 in splenocytes and OVA-specific IgE production in serum from OVA-sensitized mice. Moreover, oral administration of FP augmented CD4^+^CD25^+^ T cells and CD103^+^CD11c^+^ DCs. In animals of the FP group, the proportion of FP was increased in the mesenteric lymph nodes (MLNs), as was the proportion of Deferribacteres in the cecum. Oral administration of FP may inhibit the Th2 immune response by incorporation into the MLNs and/or by inducing changes in the gut microbiota. Thus, FP may be useful in alleviating antigen-induced Th2 immune responses.

## Introduction

In recent years, the number of individuals suffering from allergic diseases has been rising globally, which has developed into an increasingly serious issue for societies worldwide (1, 2). Allergic diseases are thought to be caused by a combination of genetic, lifestyle-related, and environmental factors. Both the activation of T-helper-2 (Th2) cell immune responses and the immune regulation of regulatory T cells (Tregs) have been implicated in the pathogenesis of allergic reactions (3–5). An allergy is an excessive immune response to a specific environmental antigen that normally is tolerated by the general population; the term also has become synonymous with hypersensitivity (6). Th2 cells are thought to play a key role in the onset of allergic reactions, given that this class of cells produces the pro-inflammatory cytokines interleukin (IL)-4, IL-5, and IL-13, and induces the differentiation of B cells into plasma cells. In response to stimulation by these cytokines, particularly IL-4, B cells undergo a class switch and differentiate into plasma cells, producing high-affinity IgE antibodies (7).

It has been reported that allergic symptoms can be alleviated by the intake of certain foods, components thereof, or probiotics such as tea (extracts of *Camellia sinensis* L.), catechin, lactic acid bacteria, and bifidobacteria (8, 9). In particularly, tea catechins, a group of polyphenolic compounds like epigallocatechin-3-*O*-(3-*O*-methyl) gallate (EGCG3”Me) and epigallocatechin-3-*O*-(4-*O*-methyl) gallate (EGCG4”Me), exert strong anti-allergic effects (10–13). Catechins constitute 30–42% of the dry weight of green tea. Major catechins are (+)-epigallocatechin gallate (EGCG), (-)-epicatechin gallate (ECG), and (-)-epicatechin (EC) (14). The majority of tea catechins are biotransformed by gut microbiota and then absorbed into the blood stream or excreted in the feces (15). The reciprocal relationship between polyphenols and gut microbiota contributes to host health benefits. However, it is unclear whether catechin-metabolizing bacteria have anti-allergic effects.

The body surface, intraoral region, gastrointestinal tract, and skin are colonized by huge numbers of different bacteria. The gut microbiota is the most abundant group, constituting more than 90% of commensal bacteria in the whole body (16). Recently, the host immune regulatory function has been shown to be regulated by the gut microbiome (17, 18). Moreover, the gut microbiota has been reported to be involved in systemic disease. For instance, the composition of the gut microbiota has been shown to be altered in infants with an allergic disease compared with that in healthy infants (19). Feehley et al. also reported that fecal transplantation from an infant with a milk allergy into germ-free mice induced anaphylactic reactions to milk in the mice (20). These reports suggest that the gut microbiota can regulate allergic immune responses. The composition of the gut microbiota also is known to be changed by factors such as the environment, lifestyle, exercise, and stress (21).

In the present study, we focused on one constituent of the gut microbiota, *Flavonifractor plautii* (FP). FP is a gram-positive anaerobic bacterium of the genus Clostridium. FP has been isolated from human feces and shown to metabolize catechins (15, 22). Atarashi et al. also reported that a mix of 17 bacterial species including FP induced the activation of Tregs, resulting in alleviation of pathology in mice with colitis and allergic disease (23). In the present study, we evaluated the anti-Th2 immune response associated with FP.

## Materials and Methods

### Reagents

Cell culture reagents and supplies were purchased from Thermo Fisher Scientific, Inc. (Waltham, MA, USA, RRID:SCR_008452). All other chemicals were purchased from Nacalai Tesque (Kyoto, Japan, RRID:SCR_013519). Antibody reagents were purchased from BioLegend (San Diego, CA, USA, RRID:SCR_001134) and Sigma-Aldrich (St. Louis, MO, USA, RRID:SCR_008988).

### Culture Conditions for FP

FP was obtained from the American Type Culture Collection (*Flavonifractor plautii* ATCC ®29863™, Rockville, MD, USA, RRID:SCR_001672). The strain was cultured in GAM broth (Nissui Pharmaceutical Co., Ltd., Tokyo, Japan) according to the medium manufacturer's protocol. The bacterial cells were pelleted by centrifugation at 8,000 × *g*, 4°C, for 5 min and then washed with and re-suspended in sterile water to yield a suspension at a density of 1 × 10^11^ colony-forming units [cfu] per mL. The resulting suspension was lyophilized and the bacterial cells were stored at −80°C until used for the experiments.

### Ovalbumin-Sensitized Mice

**Figure 1:**
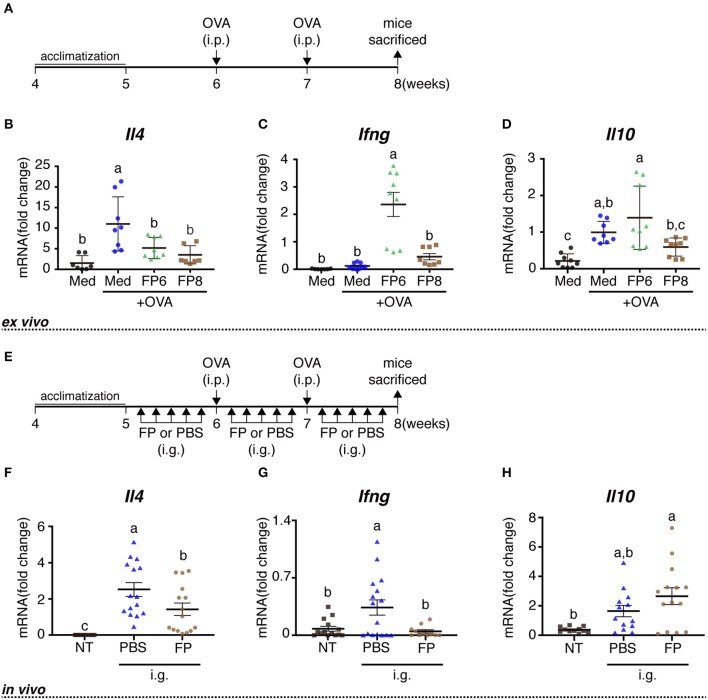
Immune effects of FP treatment or oral administration on OVA-sensitized mice. Schedule for experiments examining the immune activity of FP treatment *ex vivo* **(A)** and orally administered FP *in vivo* **(E)**. Splenocytes from OVA-sensitized mice were treated with Med (Medium), OVA (1 mg/mL ovalbumin), FP6 (1 mg/mL OVA + FP 1x10^6^ cfu/well), or FP8 (1 mg/mL OVA + FP 1 × 10^8^ cfu/well) for 24 h **(A,B)**. The expression of *Il4* **(B,F)**, *Ifng* **(C,G)**, and *Il10* **(D,H)** were measured by qPCR. Data are expressed as mean ± SD and pooled from three or four independent experiments (*n* = 7–9/group, A-D; *n* = 8–16/group, **E–H**); expression was normalized to that of the housekeeping gene encoding β-actin. No detected data were excluded. Different letters (i.e., a, b, c) indicate significant differences among groups (*p* < 0.05).

Female BALB/c mice (RRID:IMSR_APB:8492) (4 weeks of age) were purchased from Japan SLC (Hamamatsu, Japan), housed under temperature- and light-controlled conditions, and provided with *ad libitum* access to a standard diet (MF; Oriental Yeast Co. Ltd., Tokyo, Japan) and sterile water for 1 week. At 6 and 7 weeks of age, mice were sensitized by intraperitoneal (i.p.) injection with 50 μg of albumin from chicken egg white (ovalbumin, OVA, grade V; Sigma); aluminum hydroxide gel was used as an adjuvant at an allergen:adjuvant ratio of 1:50. From 8 weeks of age, OVA-sensitized mice were used for cell culture experiment (Figure 1A).

### Splenocyte Cultures

Splenocytes from OVA-sensitized female BALB/c mice (8 weeks of age) were prepared as described previously (24). Splenocytes were seeded onto 24-well plates (Nalge Nunc International K.K., Tokyo, Japan) at a density of 2 × 10^6^ cells/well in complete Roswell Park Memorial Institute (RPMI) 1640 medium (Sigma-Aldrich) supplemented with 10% fetal bovine serum, 1% non-essential amino acids, and antibiotics (100 U/mL penicillin, 100 mg/mL streptomycin), 25 mM HEPES, 1.0 mM sodium pyruvate, and 0.0035% 2-mercaptoethanol. Splenocytes then were cultured with FP (FP6: 1 × 10^6^ cfu; FP8: 1 × 10^8^ cfu) for 24 h, followed by stimulation with 1 mg/mL OVA at 37°C for 72 h.

### Oral Administration of FP into OVA-Sensitized Mice

The OVA-sensitized mice were randomly allocated into three groups: OVA-non-sensitized mice (NT group), OVA-sensitized mice orally administered with phosphate-buffered saline (PBS) (PBS group), and OVA-sensitized mice orally administered with FP (FP group). Mice in the FP group received FP (1 × 10^6^ cfu/mouse) by oral gavage once daily, five times per week, for 3 weeks from 5 to 7 weeks of age (Figure 1E). In contrast, mice in the PBS group received PBS (200 μL/mouse) by oral gavage once daily, five times per week, for 3 weeks (Figure 1E). At 8 weeks of age, mice were euthanized, and then splenocytes, mesenteric lymph nodes (MLNs), and serum from each treated mouse were used for analysis.

### *Ex vivo* Culture of Splenocytes or MLNs

Splenocytes and MLNs from OVA-sensitized female mice (8 weeks of age) were prepared as described previously (24, 25). Cells (2 × 10^6^ cells/well) were suspended in the complete RPMI1640 medium containing 1 mg/mL OVA and incubated at 37°C for 72 h in 24-well plates. Following the incubation, cells and culture supernatants were harvested by centrifugation. Cells were subjected to flow cytometric and gene expression analysis.

### T Cell-DC Co-culture Assay

CD11c^+^ dendritic cells (DCs) were prepared from splenocytes and MLNs using the MACS cell sorting system (Miltenyi Biotec, Auburn, CA, RRID:SCR_008984). DCs (2 × 10^4^~2 × 10^5^ cells/well) were suspended in complete RPMI1640 medium and were incubated at 37°C for 24 h in 96-well plates. The resulting cells were subjected to flow cytometric analysis. Naïve CD4^+^ T cells and CD11c^+^ DCs were prepared from MLNs using the MACS cell sorting system. Co-culture analysis was performed as described previously (26). Briefly, T cells and DCs were mixed at a ratio of 6:1 in complete RPMI1640 medium and incubated at 37°C for 96 h in 96-well plates. The resulting cells were subjected to flow cytometric analysis.

### Flow Cytometric Analysis

For determination of intracellular levels of IL-4, IL-10, and IFN-γ, cells were incubated at 37°C for 4 h in complete RPMI1640 medium containing 20 ng/mL phorbol-12-myristate-13-acetate, 1 μg/mL ionomycin, and 3 μg/mL brefeldin A. The stimulated cells then were fixed in 4% paraformaldehyde for 15 min at room temperature. Fixed cells were permeabilized in PBS-BSA [PBS containing 1% bovine serum albumin (BSA)] and 0.5% Triton-X100, along with one of the following fluorescently labeled antibodies: phycoerythrin (PE) -conjugated anti-Foxp3 (MF-14, RRID:AB_1089118), PE-conjugated anti-IL-4 (11B11, RRID:AB_315318), or PE-conjugated anti-IFN-γ (XMG1.2, RRID:AB_315401). Labeled isotype control antibodies consisted of PE-conjugated rat IgG1κ (RTK2071, RRID:AB_326514), biotin-conjugated rat IgG2bκ (MRG2b-85, RRID:AB_492999), Alexa Fluor® 488 (AF488) -conjugated Armenian hamster IgG (HTK888, RRID: AB_2814703), and biotin-conjugated rat IgG2aκ (RTK2758, RRID:AB_2783537). After washing with PBS-BSA and centrifugation for 5 min at 300 × *g*, 4°C. the cells were stained for analysis of surface makers by incubation for 1 h at 4°C in PBS-BSA containing allophycocyanin (APC) -conjugated anti-CD3 (17A2, RRID:AB_312668), AF488-conjugated anti-CD4 (GK1.5, RRID:AB_389302), biotin-conjugated anti-F4/80 (BM8, RRID:AB_893501), biotin-conjugated anti-CD11c (3.9, RRID:AB_313772), and AF488-conjugated anti-CD103 (2E7, RRID:AB_535949). Labeled isotype control antibodies consisted of biotin-conjugated Armenian hamster IgG (HTK888), AF488-conjugated rat IgG2bκ (RTK4530, RRID:AB_389321), AF488-conjugated Armenian hamster IgG (HTK888, RRID:AB_2814703), and biotin-conjugated rat IgG2aκ (RTK2758, RRID:AB_2783537). After washing with PBS-BSA and centrifugation for 5 min at 300 × *g*, 4°C. APC-, PE-, and PerCP/Cy5.5-conjugated streptavidin were used as linkers for the biotin-conjugated antibodies. Cells were washed and the percentage of Foxp3^+^ CD4^+^ CD3^+^ cells, IL-4^+^CD4^+^ cells, IFN-γ^+^CD4^+^ cells, and CD103^+^ CD11c^+^ DCs were determined using a Sony SH800 Cell Sorter (Sony, Tokyo, Japan) or a FACSCalibur (BD Biosciences, San Jose, CA, USA, RRID:SCR_013311). Each flow cytometer was equipped with both a krypton laser (642 nm) and an argon laser (488 nm). For each of the flow cytometric analyses, 1.0 ×10^3^~1.0 ×10^4^ events were recorded. A compensation setup including a single fluorescent cell with SH800 software was used. The data were analyzed using FlowJo (ver. 10.1r5).

### Gene Expression Analysis

Total RNA from FP- and/or OVA-stimulated splenocytes was extracted using NucleoSpin RNA Kits (TaKaRa Bio Inc., Osaka, Japan) in accordance with the manufacturer's instructions. The purified total RNA was reverse-transcribed using a PrimeScript^TM^ RT Master Mix (TaKaRa Bio Inc., Osaka, Japan) according to the manufacturer's instructions. Quantitative PCR (qPCR) reactions were performed as described previously (27). Primers for the transcripts encoding β-actin, IL-4, and IFN-γ were purchased from TaKaRa Bio, Inc.

### Relative Abundance of Bacterial Phyla by Amount of 16S rDNA

MLNs were prepared as described previously (25). At collection, cecal contents and MLNs were immersed in RNAlater (Qiagen, Valencia, CA, USA, RRID:SCR_008539) at 4°C for 24 h. The RNAlater then was removed from the cecal contents and MLNs by centrifugation at 8,000 × *g* for 5 min. Thereafter, the cecal contents were stored at −20°C until DNA extraction. At the time of DNA extraction, the cecal contents (5–10 mg) and MLNs were mixed with the Inhibitory Ex buffer (200 μL) of the QIAamp Fast DNA Stool Mini Kit (Qiagen, RRID:SCR_008539) in a Zirco Prep Mini tube (Nippon Genetics Co., Ltd., Tokyo, Japan). The sample was shaken for 5 min using a Bug Crasher μT-12 (Taitec Co., Saitama, Japan). DNA was purified from the mixture using the QIAamp Fast DNA Stool Mini Kit (Qiagen, RRID:SCR_008539). qPCR was performed using the KAPA SYBR FAST qPCR master mix (KAPA Biosystems). The primer sequences are provided in Table 1. The 16S rDNA levels of the gut microbiota in the MLNs and cecal contents were quantified by qPCR with a Thermal Cycler Dice Real Time System II (TaKaRa Bio, Inc.) and the following cycling conditions: 95°C for 10 s, followed by 50 cycles of 95°C for 5 s and 60°C for 30 s. Dissociation was performed by incubation at 95°C for 15 s, 60°C for 30 s, and 95°C for 15 s. Quantification of the target 16S rDNA levels of Firmicutes, Bacteroides/Prevotella, β/γ/δ/ε-Proteobacteria, Actinobacteria, Deferribacteres, Verrucomicrobia, Tenericutes, and FP in cecal contents was performed using the ΔΔCt method with normalization to the total 16S rDNA content (31).

**Table 1:** 16S rDNA gene-specific primers.

**Target group**	**Primer name**	**Sequence (5^**′**^ to 3^**′**^)**	**References**
Bacteroides/Prevotella	F_Bacter	CCTWCGATGGATAGGGGTT	(28)
	R_Bacter	CACGCTACTTGGCTGGTTCAG	
Firmicutes	Firm350f	GGCAGCAGTRGGGAATCTTC	(29)
	Firm841r	ACACYTAGYACTCATCGTTT	
Actinobacteria	Act664F	TGTAGCGGTGGAATGCGC	(30)
	Act941R	AATTAAGCCACATGCTCCGCT	
Deferribacteres	Defer1115F	CTATTTCCAGTTGCTAACGG	(30)
	Defer1265R	GAGHTGCTTCCCTCTGATTATG	
Verrucomicrobia	Ver1165F	TCAKGTCAGTATGGCCCTTAT	(30)
	Ver1263R	CAGTTTTYAGGATTTCCTCCGCC	
Tenericutes	Ten662F	ATGTGTAGCGGTAAAATGCGTAA	(30)
	Ten862R	CMTACTTGCGTACGTACTACT	
β-proteobacteria	Beta979F	AACGCGAAAAACCTTACCTACC	(30)
	Beta1130R	TGCCCTTTCGTAGCAACTAGTG	
ε-proteobacteria	Epslion940F	TAGGCTTGACATTGATAGAATC	(30)
	Epsloin1129R	CTTACGAAGGCAGTCTCCTTA	
δ/γ-proteobacteria	Gamma877F	GCTAACGCATTAAGTRYCCCG	(30)
	Gamma1066R	GCCATGCRGCACCTGTCT	
All bacteria	8f_All	GRGTTYGATYMTGGCTCAG	(28)
	340R	ACTGCTGCCTCCCGTAGGAGT	
*Flavonifractor plautii*	FP_F	TGAGTAACGCGTGAGGAACC	This study
ATCC29863^T^	FP_R	TCGTCGGGTACCGTCATTTG	

*Nucleotides symbols: R = A or G; Y = C or T; W = A or T; M = A or C; K = T or G*.

### ELISA

Commercially available ELISA kits were used to quantify serum levels of OVA-specific IgE (eBioscience Inc., San Diego, CA, USA, RRID:SCR_003660) and lipopolysaccharide binding protein (LBP) (Biometec, Greifswald, Germany). The concentrations of IL-10 in culture supernatants was measured using a Mouse IL-10 IQELISA™ Kit (RayBiotech, Norcross, GA, USA). These ELISAs were performed according to the respective manufacturer's instructions.

### Western Blotting

The flash-frozen MLN samples were thawed and suspended in CelLytic M (Sigma). The resulting lysates then were diluted in sample buffer (FUJIFILM Wako Pure Chemical Co.) and incubated at 70°C for 30 min. Samples were resolved by 15% (v/v) SDS-PAGE. Proteins then were transferred from the gel onto a Hybond-P PVDF membrane (GE Healthcare Japan, Tokyo, Japan). After blocking for 1 h, the membrane was incubated overnight with anti-mouse Foxp3 (SAB2700362), anti-mouse GATA3 (SAB1405059), and anti-mouse β-actin (clone AC-15) antibodies at 4°C, followed by incubation with a secondary antibody. The immunoblots were visualized using the ECL Prime Western Blotting Detection Reagent (GE Healthcare Japan). The protein bands were detected using an ImageQuant LAS 4000mini chemiluminescence analyzer (GE Healthcare Japan) and analyzed using ImageJ software (version 1.51; National Institutes of Health, Bethesda, MD, USA, RRID:SCR_003070). Quantitation of the proteins was performed by densitometry; values were normalized to the expression level of the housekeeping protein β-actin.

### Statistical Analysis

Statistical analyses were performed using Prism 7 (GraphPad Software, Inc., San Diego, CA, USA, RRID:SCR_002798). The significance of differences among 3 or more groups were assessed by two-tailed one-way analysis of variance (ANOVA) followed by the Tukey–Kramer test. Differences were considered significant at *p* < 0.05.

## Results

### FP Ameliorates Th2 Immune Responses

**Figure 2:**
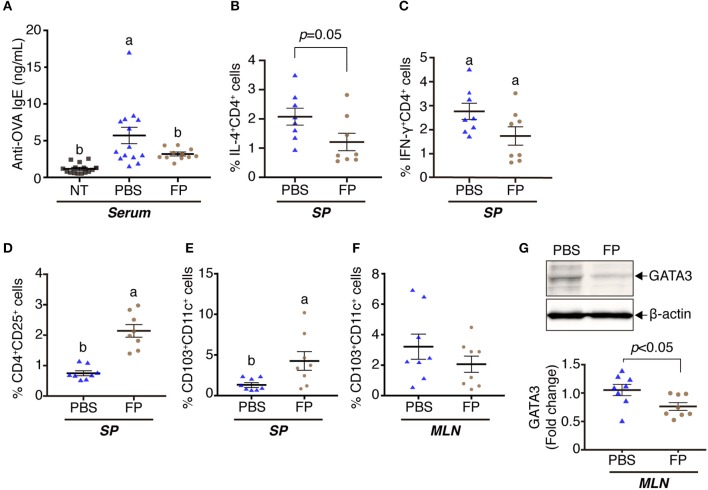
Oral administration of FP augmented immune regulatory effects on OVA-sensitized mice. The levels of anti-OVA-specific IgE were measured in serum by ELISA **(A)**. IL-4^+^CD4^+^ **(B)**, IFN-γ^+^CD4^+^ **(C)**, CD4^+^CD25^+^ **(D)**, and CD103^+^CD11c^+^ **(E,F)** cells among the splenocytes **(B–E)** or MLNs **(F)** were analyzed by flow cytometry. GATA3 were determined by western blotting **(G)**. Data are expressed as mean ± SE and pooled from four independent experiments (*n* = 8–16/group). Different letters (i.e., a, b, c) indicate significant differences among groups (*p* < 0.05).

In an initial set of experiments, we examined the effects of OVA and FP (at 1 ×10^6^ or 1 ×10^8^ cfu/well) on transcription, as assessed *in vitro* using splenocytes derived from OVA-sensitized mice. Exposure of cells to OVA alone yielded significant increases in the levels of *Il4* and *Il10*, but not in that of *Ifng*, compared to the effect of medium lacking antigen (Figures 1B–D). Exposure of cells to OVA and FP (at 10^6^ or 10^8^ cfu/well) yielded significant attenuation of the OVA-induced accumulation of *Il4* but not that of *Il10*. Indeed, OVA plus the lower dose of FP potentiated the level of *Ifng* seen with OVA alone, an effect not seen with the higher dose of FP (Figure 1C). In a second set of experiments, we extended this analysis to the *in vivo* response of OVA-sensitized mice. Notably, animals administered PBS demonstrated significant accumulation of *Il4* and *Ifng* transcripts (but not of *Il10*) (Figures 1F–H) and significant decreases of anti-OVA IgE in serum (Figure 2A). Moreover, we found that FP decreased the numbers of IL-4^+^CD4^+^, IFN-γ^+^CD4^+^ cells (Figures 2B,C) and increased CD4^+^CD25^+^ T cells, CD103^+^CD11c^+^ DCs in spleen (Figures 2D,E). CD103^+^CD11c^+^ DCs in the MLNs of the FP group did not differ significantly from that in the PBS group (Figure 2F). However, the expression of GATA3 in the MLNs of the FP group was significantly decreased compared with the PBS group (Figure 2G).

### FP Reaches MLNs Following Oral Administration

**Figure 3:**
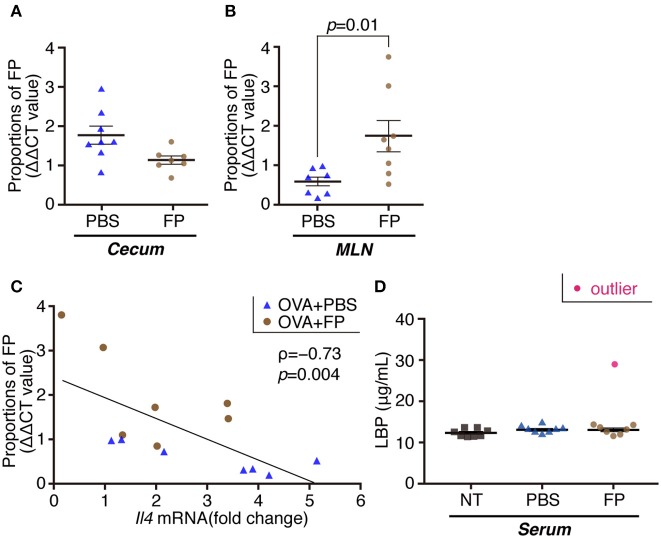
Proportions of FP composition of cecal contents/MLNs and the levels of serum LBP. Proportions of FP were determined by qPCR in cecal contents **(A)** and MLNs **(B)**. Correlation between *Il4* expression in splenocytes and FP composition of MLNs **(C)**. ρ indicates Spearman's correlation coefficient; and *p* indicates *p* value (*n* = 7) **(C)**. The levels of serum LBP were determined by ELISA **(D)**. Data are expressed as mean ± SE and pooled from two independent experiments (*n* = 7–8/group). There were significant differences between PBS and FP groups (*p* = 0.01).

**Figure 4:**
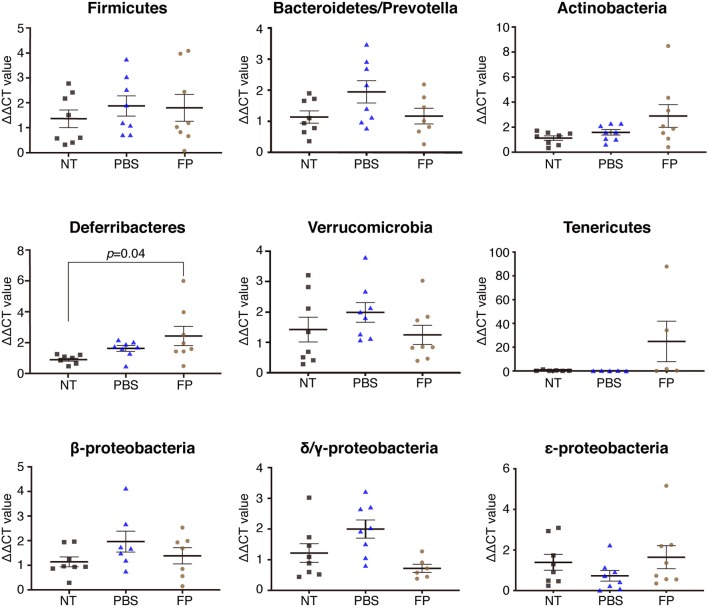
Effect of FP on the cecal microbiota. Proportions of Firmicutes, Bacteroides/Prevotella, Actinobacteria, Deferribacteres, Verrucomicrobia, Tenericutes, β-Proteobacteria, δ/γ-Proteobacteria, and ε-Proteobacteria were assessed in cecal contents by qPCR. Data are expressed as mean ± SE and pooled from two independent experiments (*n* = 5–8/group).

We next examined the destination of FP following oral administration, and effects of the bacteria on the gut microbiota. The proportion of FP in the MLNs of the FP group was increased significantly compared with that in the PBS group (Figure 3A). In contrast, the proportion of FP in the cecal contents did not differ significantly between the FP and PBS groups (Figure 3B). It is formally possible that FP gained access to the MLNs in these animals by means of damage to the intestinal barrier. The expression of *Il4* in spleen showed a significant negative correlation with the relative abundance of FP in the MLNs when comparing between the FP and PBS groups (ρ = −0.73, *p* = 0.004; Figure 3C). To address this issue, we assessed the state of the intestinal barrier in these mice by measuring the serum level of lipopolysaccharide-binding protein (LBP). Secreted LBP would affect intestinal barrier against bacteria. Notably, we detected no significant difference in the serum LBP levels between the FP and PBS groups (Figure 3D). The proportion of Deferribacteres in the cecal contents of the FP group was increased significantly compared with that in the NT group (Figure 4). We found no significant differences for any of the other comparisons. These results suggested that FP did not affect the intestinal barrier function, indicating that FP (or at least some FP genomic DNA) may reach the MLNs.

### FP Induces CD103^+^ DC and Foxp3^+^ T cell Differentiation

**Figure 5:**
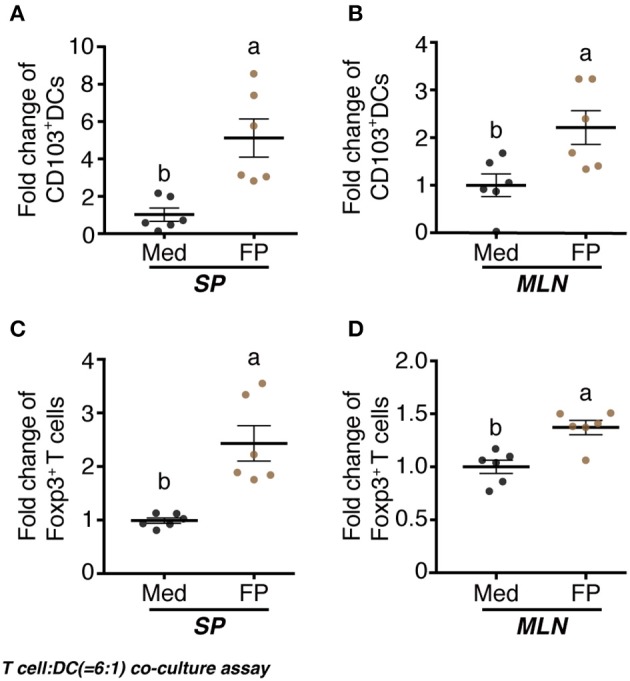
Effect of FP on T cell-DC (6:1) co-culture assay. naïve CD4^+^ T cells and CD11c^+^ DCs were prepared from splenocytes and MLNs using the MACS cell sorting system. DCs were treated with Med or FP (FP 1 ×10^8^ cfu/well) at 37°C for 24 h in 96-well plates. T cells and DCs were mixed at a ratio of 6:1 and treated with Med or FP at 37°C for 96 h in 96-well plates. CD103^+^ DCs **(A,B)** and Foxp3^+^ T **(C,D)** cells in splenocytes **(A,C)**, or MLNs **(B,D)** were analyzed by flow cytometry. Data are expressed as mean ± SD and pooled from two independent experiments (*n* = 6/group). Different letters (i.e., a, b) indicate significant differences among groups (*p* < 0.05).

We examined the differentiation of CD103^+^ DCs by FP. Significantly increased fold-changes in the numbers of CD103^+^ DCs among the splenocyte DCs and MLN DCs were observed in the FP group compared with those in the Med group (Figures 5A,B). Additionally, we examined the differentiation of Foxp3^+^ T cells by FP. Again, significantly increased fold-changes in the numbers of Foxp3^+^ T cells among the splenocyte T cells and MLNs T cells were observed in the FP group (Figures 5C,D).

## Discussion

The FP species has been classified into *Clostridia* cluster IV in the Ruminococcaceae family. Several reports have shown that the gut microbiota of *Clostridia* cluster IV is capable of inhibiting inflammation. For instance, Li et al. reported that *Clostridium leptum* induces proliferation of Treg cells (32); Rossi et al. reported that *Faecalibacterium prausnitzii* (a member of the Ruminococcaceae family) strongly induces IL-10 production (33); and Wu et al. reported that decreases in the proportion of the *Ruminococcaceae* family induce IgE-associated eczema in infants (34). The results of the present study showed that Th2 immune responses induced by the addition of OVA were attenuated significantly by the ingestion of FP in OVA-sensitized mice. Thus, our work suggested that FP influences the balance of the host immune system.

*In vitro*, the expression of mRNAs encoding IFN-γ (a Th1 cytokine) and IL-10 (a regulatory cytokine) was increased by FP, effects that were not seen for expression of the gene encoding IL-4 (a Th2 cytokine). These results suggested that FP inhibits Th2 responses by activating Treg and Th1 cells. Indeed, it has been reported that Tregs are induced and Th2 responses are suppressed under conditions that lead to increases in the number of CD103^+^ DCs that are tolerant to the gram-positive bacteria *Bifidobacterium* and *Lactobacillus* (*L*.) *rhamnosus* GG as well as to the FP strain (35, 36). The present study revealed that FP induces CD103^+^ DC differentiation *in vitro*. However, mice administered FP did not exhibit an increase in CD103^+^ MLN DCs. Activated-DCs move to the MLN via Peyer's patches and induce T cell differentiation. The differentiated T cells are subsequently involved in the systemic immune response (37). In this study, the MLN was collected from mice 48 h after FP administration. The *in vivo* experiment does not simulate the *in vitro* experiment with the direct stimulation by FP. In other words, the CD103^+^ DC differentiation phenomenon with FP administration will need to be tested using more detailed time monitoring. *In vivo, Il10* expression and the population of CD4^+^CD25^+^ cells in splenocytes was increased in the FP group. Foxp3, Treg transcription factor, has been reported to be specifically expressed on CD4^+^CD25^+^ T cells and is used as a cell surface marker for Tregs (38, 39). CD4^+^CD25^+^ T cells have been reported to contribute to the alleviation and prevention of allergic diseases (40, 41). Orally administered FP strains may reach the intestinal tract and stimulate intestinal cells. A number of Treg cells are present in the intestinal tract and contribute to the induction of tolerance (42). FP has been reported to increase the number of Treg cells in colonic tissues and to exert anti-allergic effects (23). Indeed, we found that FP induces Foxp3^+^CD4^+^ T cell differentiation *in vitro*. We also showed that FP decreases the expression of GATA3, a transcription factor for Th2 cells, in the MLNs of OVA-sensitized mice. It will be important to investigate how the oral administration of FP downregulates the Th2 immune response through DC and/or Treg responses in the MLN. Some reports have established that immune cell transplantation plays a fundamental role in animal physiology (43–45). Further studies will be necessary to confirm whether FP-treated DCs/Tregs downregulate the Th2 immune response by inhibiting the differentiation of Th2 cells. We infer that oral administration of FP and induction of Tregs dominates the intestinal immune response, and is followed by migration of FP through the bloodstream and lymphatic system, where the bacteria contribute to the splenic tissue immune response. However, in the present study, *in vivo Ifng* levels differed from those in the *in vitro* study. In addition, immune responses appeared to be decreased in the *in vivo* experiments compared to those in the *in vitro* experiments. Similar results were obtained in the report of Tobita et al. using *L. crispatus* (46). This phenomenon may reflect differences in the environments in which the bacteria act. The spleen cells used in the *in vitro* study were already OVA-sensitized, meaning that the Th2 cells already had been activated. Therefore, Th1 and Treg presumably were activated by the immunosuppressive effects of the FP strain, restoring the T cell balance to normal. On the other hand, since the FP was administered prior to OVA sensitization in the *in vivo* test, the immunosuppressive effect of FP on Tregs would predominate in that experiment, preventing Th2 activation without Th1 activation.

Oral administration of *L. reuterii*, a gram-positive bacterium similar to FP, in a mouse allergy model has been reported to decrease *Il4* and *Ifng*, and increase *Il10* mRNA expression in the spleen (47). Moreover, FP may be responsible for suppressing allergic responses by improving intestinal barrier function. Based on the above results, we infer that FP contributes to control of the allergic response by activating Tregs and suppressing the expression of *Il4*, a Th2-related gene. In the future, to confirm these mechanistic differences, it will be necessary to test the immune status of mice treated with the FP strain prior to OVA sensitization. Kwon et al. reported that oral administration of *L. sakei* WIKIM30 induces tolerogenic DCs and Treg cells, thereby improving allergic symptoms in mouse models of allergic dermatitis (48). It also has been reported that oral administration of Bifidobacterium increases Treg numbers and improves allergic symptoms in OVA-sensitized allergy model mice (49, 50).

This study showed that the serum titer of OVA-specific IgE was decreased significantly by the intake of FP. In contrast, there was no significant difference (between the FP and PBS groups) in the expression of genes encoding proteins (IL-7 and IL-21) related to class switching (51, 52) (Figure S1). Moreover, no significant differences in the levels of SCFAs were detected between the FP and PBS groups, in either the cecal contents or sera of these mice (Figure S2). Thus, oral administration of FP did not yield a significant change in SCFA production. These results suggested that the SCFAs do not mediate Th2 suppression by FP. Thus, FP may not be involved in the IgE class-switch recombination. Accordingly, FP may be involved in the inhibition of OVA-specific IgE by regulating the Th2 immune response. The expression of *Il4* in splenocytes showed a significant negative correlation with the relative abundance of FP in the MLNs when comparing between the FP and PBS groups. Accordingly, the accumulation of FP in the MLNs may regulate the Th2 immune response. The proportion of Deferribacteres in the cecal contents of the FP group was increased significantly compared to that in the NT group. This observation may relate to a report by Ma et al. indicating that the proportion of Deferribacteres was increased following oral administration of probiotics in an OVA-induced mouse model of food allergy (53). Deferribacteres bacteria are able to obtain energy through obligate or facultative anaerobic metabolism. The iron metabolism of Deferribacteres in intestinal flora has been shown to be related to iron balance in the gastrointestinal tract (54). By analogy, we infer that FP also may be related to iron metabolism.

In conclusion, the collection of FP in MLNs may be involved in inhibition of the Th2 immune response. Our results demonstrated that FP mediates the inhibition of OVA-specific IgE production in a Th2-dominant environment. Given that allergy is closely associated with the production of antigen-specific IgE, FP may be useful in alleviating antigen-specific Th2 immune responses in a Th2-dominant environment. The results described here suggest that FP might be employed as a probiotic in the treatment of allergy. However, other work has suggested this organism as a possible causative agent of acute cholecystitis in a patient with a bloodstream infection (55). Clearly, further studies addressing the safety of FP will be needed before this bacterium can be employed as an anti-allergy probiotic.

## Data Availability Statement

All datasets generated for this study are included in the article/Supplementary Material.

## Ethics Statement

All experimental procedures were carried out in accordance with the Regulations for Animal Experimentation of Shinshu University. All experimental procedures were reviewed by the Committee for Animal Experiments of Shinshu University and found to be compliant with national regulations and guidelines, as specified by Law No. 105 and Notification No. 6. Therefore, the animal protocol was approved by the Committee for Animal Experiments of Shinshu University as Approval No. 290074.

## Author Contributions

TO, YY, SS, TSa, and TSh conceived and designed the experiments. TO, YY, and AM conducted the experiments and performed mathematical analyses. TO, YY, and TSh wrote the paper. TSh designed and supervised the work. All authors reviewed and approved the manuscript.

### Conflict of Interest

The authors declare that the research was conducted in the absence of any commercial or financial relationships that could be construed as a potential conflict of interest.

## Supplementary Material

Figure S1 *Il7* and *Il21* response to FP administration. Expression of mRNAs encoding IL-7 **(A)** and IL-21 **(B)** were assessed in splenocytes isolated from OVA-sensitized mice. Data are expressed as mean ± SE of two independent experiments (*n* = 12–16).

Figure S2 Short-chain fatty acid levels in cecum and serum. Short-chain fatty acid (SCFA) analysis was performed using a high-performance liquid chromatography (HPLC) system according to the method. SCFAs were separated using a Shimadzu HPLC system. An Aminex HPX-87H column (Bio-Rad Laboratories, Hercules, CA, USA, RRID:SCR_008426) was used at 35°C. Absorbance at 210 nm was monitored to detect SCFAs using a Shimazu SPD-10A UV–Vis detector. Levels of acetate **(A)**, butyrate **(B)**, isovalerate **(C)**, propionate **(D,H)**, isobutyrate **(E,I)**, lactate **(F,J)**, and succinate **(G,K)** were measured in cecum contents **(A–G)** and serum **(H–K)** by HPLC. Data are expressed as mean ± SE of two independent experiments (*n* = 5–8). Outliers and no detected data were excluded.
