# Supplementary figures and images for: Oral Administration of Flavonifractor plautii Strongly Suppresses Th2 Immune Responses in Mice
Source: Front Immunol. 2020 Feb 28;11:379. doi: 10.3389/fimmu.2020.00379 (PMC7058663; doi:10.3389/fimmu.2020.00379)

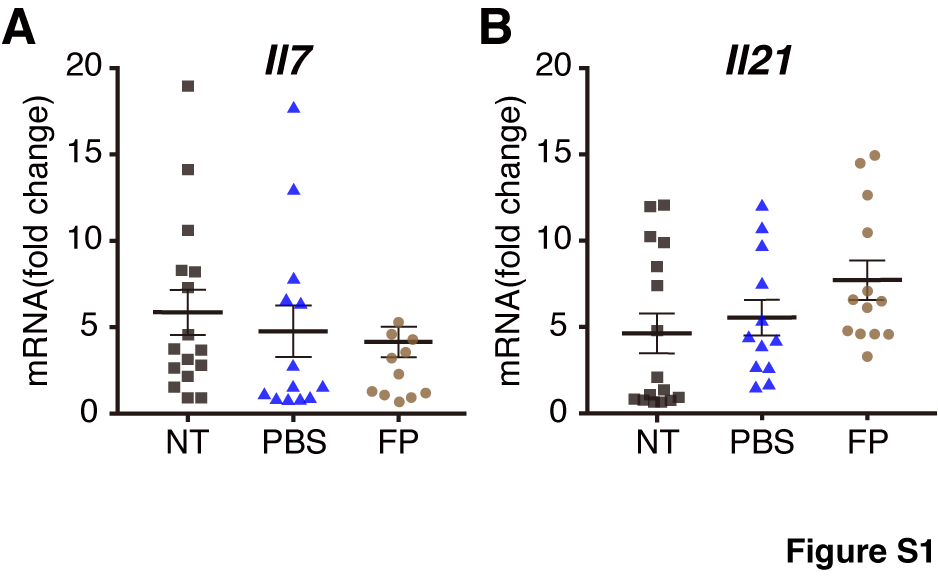

Supplement: Figure S1 — Il7 and Il21 response to FP administration. Expression of mRNAs encoding IL-7 (A) and IL-21 (B) were assessed in splenocytes isolated from OVA-sensitized mice. Data are expressed as mean ± SE of two independent experiments (n = 12–16). [file Image_1.tif]

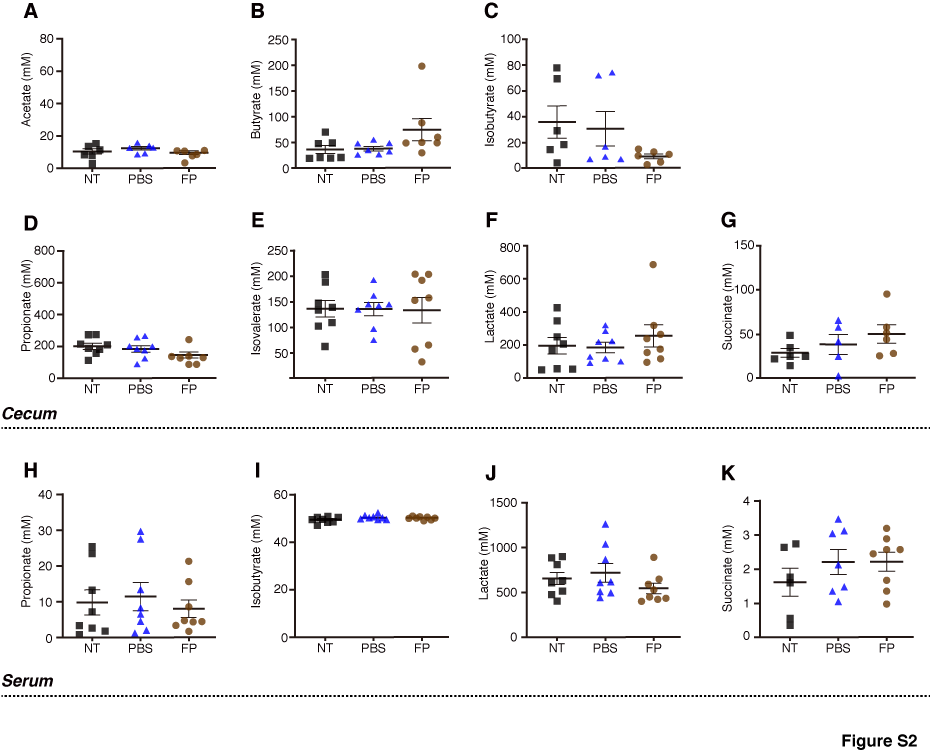

Supplement: Figure S2 — Short-chain fatty acid levels in cecum and serum. Short-chain fatty acid (SCFA) analysis was performed using a high-performance liquid chromatography (HPLC) system according to the method. SCFAs were separated using a Shimadzu HPLC system. An Aminex HPX-87H column (Bio-Rad Laboratories, Hercules, CA, USA, RRID:SCR_008426) was used at 35°C. Absorbance at 210 nm was monitored to detect SCFAs using a Shimazu SPD-10A UV–Vis detector. Levels of acetate (A), butyrate (B), isovalerate (C), propionate (D,H), isobutyrate (E,I), lactate (F,J), and succinate (G,K) were measured in cecum contents (A–G) and serum (H–K) by HPLC. Data are expressed as mean ± SE of two independent experiments (n = 5–8). Outliers and no detected data were excluded. [file Image_2.tif]
